# Case Report: A case of cervical burkitt lymphoma and literature review

**DOI:** 10.3389/fonc.2025.1696219

**Published:** 2026-01-05

**Authors:** Sen Miao, Yue Bai, Hua Hao

**Affiliations:** 1 Department of Pathology, Affiliated Hospital of Jining Medical University, Jining, China; 2 Department of Pathology, Yangpu Hospital, School of Medicine, Tongji University, Shanghai, China

**Keywords:** burkitt’s lymphoma, chemotherapy, EBER, MYC, prognosis

## Abstract

Primary cervical Burkitt’s lymphoma (BL) is an extremely rare malignancy. This report describes the case of a 55-year-old woman admitted to the hospital with irregular vaginal bleeding. Histologically, the tumor cells demonstrated infiltrative growth, relatively uniform basophilic cytoplasm. Immunohistochemical analysis revealed positivity for B-cell markers (CD20, CD79α, etc.), with MYC expression in approximately 80% of cells, fluorescence *in situ* hybridization staining showed positivity for MYC translocation and molecular testing confirmed IG rearrangement. The diagnosis of Burkitt’s lymphoma is based on its distinctive histological morphology in combination with immunohistochemistry and molecular testing.

## Introduction

Burkitt’s lymphoma (BL) is a very fast-growing and highly aggressive B-cell non-Hodgkin’s lymphoma (NHL) that can involve/affect multiple organ systems throughout the body (1). The World Health Organization (WHO) classifies BL into three clinical subtypes: endemic, sporadic, and immunodeficiency-related. The three subtypes are histologically identical and are defined primarily on the basis of their long-term distribution patterns in different geographic settings as well as the immune status of the patient (2). Sporadic lymphomas most commonly involve the abdomen and pelvis (1). The endemic type is associated with malaria or the Epstein-Barr virus (EBV). Immunodeficiency-associated BL is seen predominantly in HIV-infected patients, but may also occur rarely in organ transplant recipients and patients with innate immunodeficiencies (1). According to the WHO classification of the Haematolymphoid Tumours (5th Edition), however, Burkitt’s lymphoma is stratified into Epstein-Barr virus (EBV) -positive and EBV-negative subgroups, which represent distinct biological entities in the current molecular era.

## Case description

**Figure 1:**
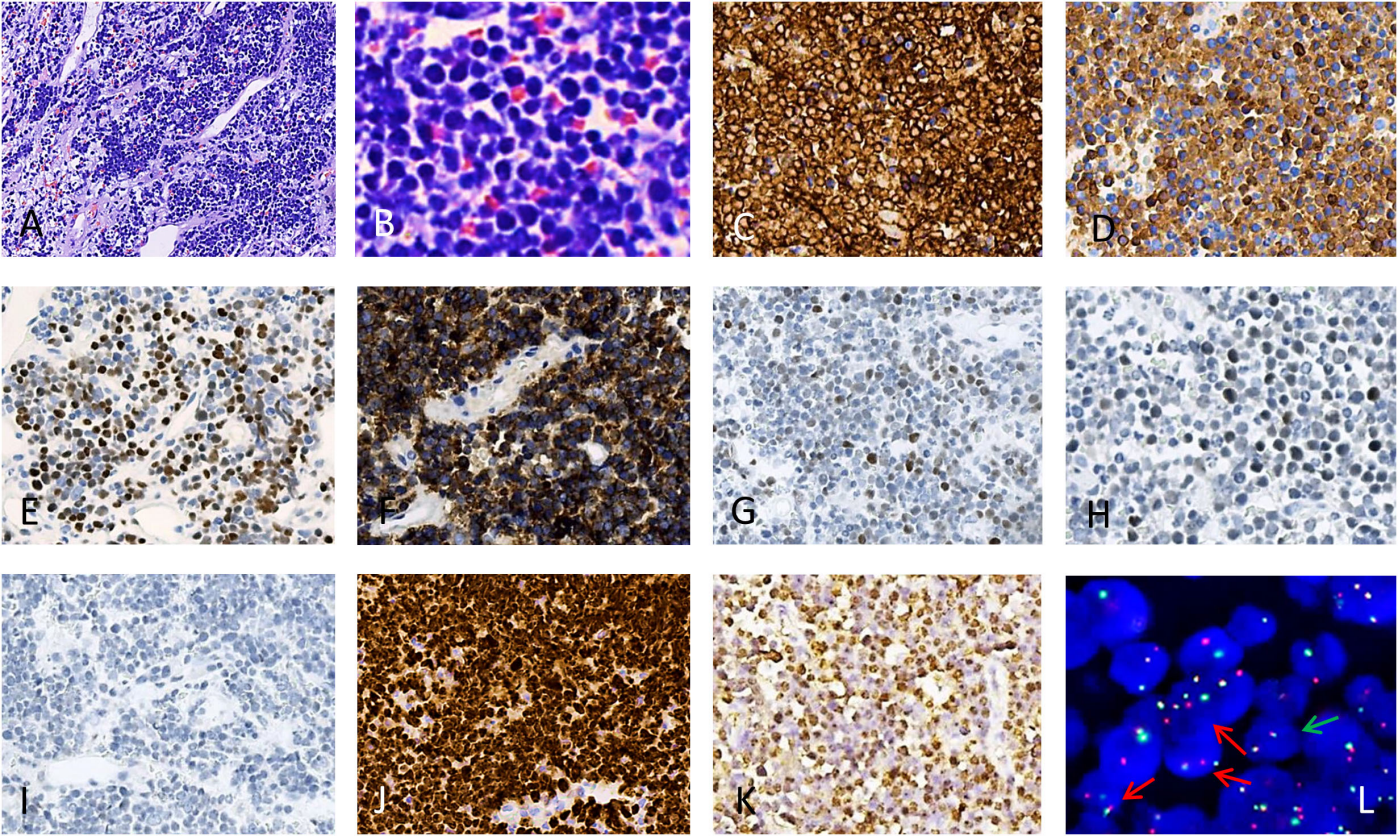
Burkitt’s lymphoma in biopsy. **(A)**, hematoxylin-eosin, × 40. **(B)**, hematoxylin-eosin, × 400. Immunohistochemical staining showed positivity for CD20 **(C)**, positivity for CD79α **(D)**, positivity for C-MYC **(E)**, positivity for CD10 **(F)**, focal positivity for BCL-6 **(G)**, focal positivity for Mum-1 (10% of cells) **(H)**, negativity for BCL-2 **(I)**, Immunohistochemical staining showed nearly 100% positivity for Ki-67 **(J)**, *in situ* hybridization staining showed positivity for EBER **(K)**, fluorescence *in situ* hybridization staining showed positivity for MYC translocation **(L)**, marking the cells harboring the MYC translocation with red arrows and the normal cell with green arrow.

A 55-year-old woman visited our hospital with postmenopausal vaginal bleeding persisting for 3 months. She had no history of surgery or trauma and reported no systemic symptoms such as fever or weight loss. Moreover, she had no history of HIV infection or any history of immunodeficiency or immunosuppression. Gynecological examination revealed an ulcerated cervical lesion measuring approximately 10 cm×10 cm, which bled upon contact. Complete Blood Count Results (May 10th, 2024): RBC count: 3.42 × 10¹²/L; WBC count: 7.3 × 10^9^/L; Hemoglobin: 97 g/L. We obtained some data from the external hospital: iron ion concentration (June, 26th, 2024) was 7.53 μmol/L (Normal range: 9-27 μmol/L), which implied microcytic hypochromic anemia. Therefore, the causes of the patient’s anemia were: iron deficiency anemia (due to blood loss) and inflammatory anemia (inflammation impairs iron utilization). Furthermore, bleeding associated with cervical tumors also contributes to the patient’s anemia. The lactatedehydrogenase (LDH) level was 426 U/L (Normal range: 109-245 U/L) (May 10th, 2024). A biopsy was performed, and the specimen was sent for pathological examination. Pathological examination revealed a grayish-white to grayish-brown soft mass (approximately 1.5 cm× 1 cm× 0.5 cm). Microscopic findings: After HE staining and microscopic examination, tumor cells exhibited a mosaic-like arrangement, with some cells showing cytoplasmic constriction and well-defined borders. Nuclei were round, and cytoplasm was deeply basophilic, revealing a starry sky phenomenon (**Figures 1A, B**). Immunohistochemical results showed that tumor cells were diffusely positive for CD20 (**Figure 1C**); positive for CD79α (**Figure 1D**); MYC (+, approximately 80%) (**Figure 1E**); CD10 (**Figure 1F**); focal moderately positive for Bc1-6 (**Figure 1G**), focal positive for Mum-1 (10% of cells) (**Figure 1H**); negative for CK, CD3, CD5, CD30, BCL-2 (**Figure 1I**); and strongly positive for Ki-67 (nearly 100%) (**Figure 1J**). *In situ* hybridization confirmed EBER positivity (**Figure 1K**). Fluorescence *in situ* hybridization staining showed positivity for MYC translocation (**Figure 1L**), and 100 cells were counted, with 71 being positive. The number of positive cells accounted for 71% of all cells. The FISH probe is a breakage probe. The probe supplier was Wuhan Kanglu Biotechnology Co., Ltd., with the catalog number: FP-243-1. Molecular testing results (monoclonal gene rearrangement testing) showed: IGH gene rearrangement was detected. The pathological diagnosis: Based on BCL-2 expression being completely negative, together with the germinal center phenotype, very high Ki-67, and the presence of MYC rearrangement, the pathological diagnosis was aggressive B-cell lymphoma of the cervix, consistent with Burkitt’s lymphoma. PET-CT findings from an external hospital on May 28, 2024: (i) An irregular soft tissue mass with high FDG uptake was noted in the cervix, measuring 10.8 × 8.7 cm, with SUVmax 18.1. The lesion involves the vagina and shows indistinct borders with the posterior bladder wall, anterior rectal wall, and pelvic bowel. The uterine body exhibits an irregular shape, with a nodule appearing to protrude beyond the uterine body. No significant abnormal FDG uptake was observed. (ii)A nodule with high FDG uptake was identified in the right adnexal region, measuring approximately 3.9 × 4.2 cm, with SUVmax 20.3, and this lesion was considered an additional site of lymphoproliferative involvement. Additionally, multiple lymph nodes with moderate FDG uptake were noted in right cervical region 1, the right axilla, and bilateral pelvic walls. The largest lymph node, located in the right axilla, measured approximately 0.9 × 1.3 cm, with SUVmax 4.7. (iii) the spleen was of normal size. Bone marrow biopsy showed no evidence of lymphoproliferative involvement. The bone marrow puncture specimen did not undergo IGH clonal detection; only a bone marrow biopsy was performed. Moreover, no tumor cells were detected in the cerebrospinal fluid. ECOG score was 3 because the patient has only partial functional capacity in daily living activities but has lost the ability to work. They can get out of bed and engage in activities for at least half of the day. Burkitt Lymphoma International Prognostic Index (BL-IPI) was 2 because the patient was over 40 years old and ECOG score was 3. Based on that lymph nodes on both sides of the diaphragm were involved and the absence of bone marrow involvement, the patient’s Ann Arbor staging was IVEX.

**Figure 2:**
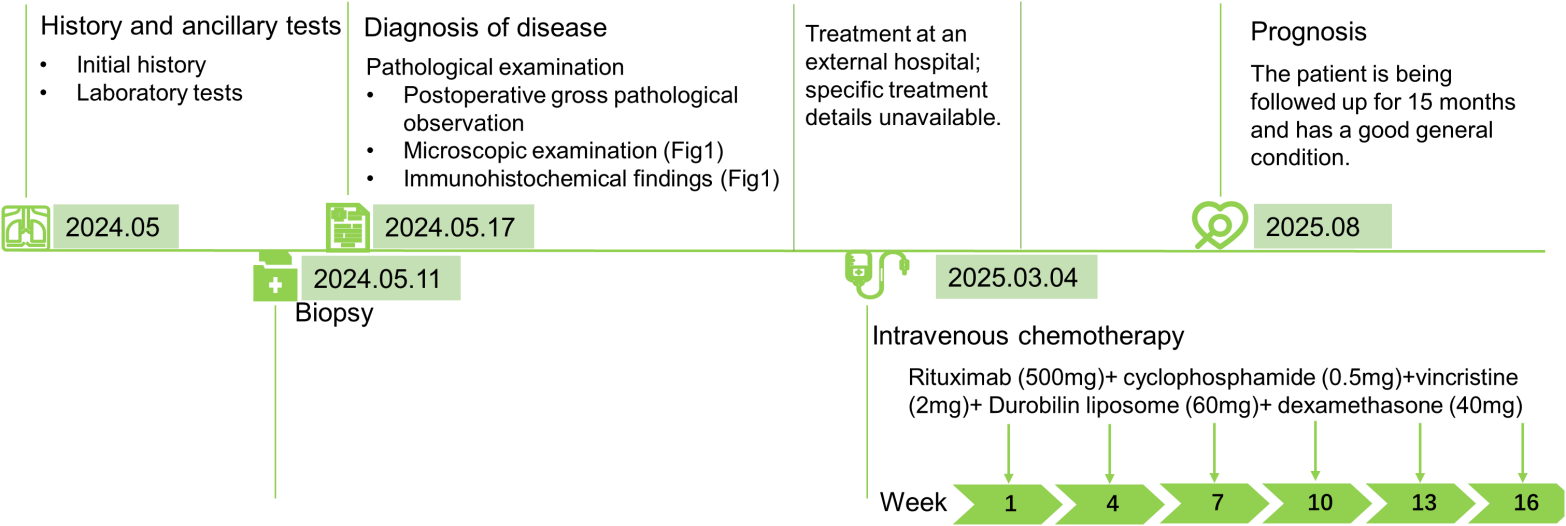
A timeline figure summarising the case diagnosis and treatment pathway.

The patient was diagnosed at our hospital in May 2024. After discharge, the patient received treatment at another hospital, though the specifics are unknown. Furthermore, the exact time interval between diagnosis confirmation and treatment initiation could not be obtained. In May 2025, the patient returned to our hospital for continued treatment, undergoing a total of six cycles of R-hyperCVAD chemotherapy and was currently in good condition. The patient’s symptoms had resolved, and the mass had disappeared. Moreover, PET-CT findings from the external hospital on Feb 10, 2025: No abnormal radioactive uptake was observed in the bilateral adnexal regions. The lactatedehydrogenase (LDH) level was 206 U/L (August 13th, 2025). Based on imaging and clinical criteria, the patient achieved complete remission (CR). A timeline summarizing the case diagnosis and treatment pathways is shown in **Figure 2**.

## Discussion

Primary lymphoma of the female genital tract is rare, accounting for only 0.2%–1.1% of all lymphomas (3). The ovary (37%) is the most common site of occurrence, followed by the cervix (21.4%) (3). The majority of the pathological types are non-Hodgkin lymphomas, with B-cell lymphomas predominating, particularly diffuse large B-cell lymphoma (DLBCL). Burkitt’s lymphoma, however, is extremely uncommon (4). Cervical involvement by Burkitt’s lymphoma is even rarer; a review of the literature over the past decade identified only three reported cases (5–7) (**Table 1**). According to the World Health Organization (WHO) HAEM5 classification criteria, Burkitt lymphoma is defined as an aggressive mature B-cell neoplasm composed of medium-sized cells with a germinal center phenotype (CD10+, BCL6+, BCL2-), a high proliferative index (Ki-67>95%), and IG::MYC juxtaposition (8). Previously, it was classified based on epidemiological characteristics into “endemic,” “non-endemic or sporadic,” and “immunodeficiency-associated.” In the current molecular era, however, Burkitt’s lymphoma is stratified into Epstein-Barr virus (EBV) -positive and EBV-negative subgroups, which represent distinct biological entities. This molecular classification overcomes the limitations of epidemiology and geography-based models and accurately reflects the essence of the disease (8). Approximately 30% of sporadic BL cases test positive for EBV and EBV infection plays a crucial role in the early stages of Burkitt’s lymphoma development by enabling B cells to evade apoptosis. Its pathogenesis exhibits a dual nature mechanism, virus-driven and mutation-driven mechanisms, that varies depending on the status of EBV infection and involving mutations in multiple key pathways (8, 9). The WHO classification of the Haematolymphoid Tumours (5th Edition) recommends distinguishing EBV-positive and EBV-negative Burkitt’s lymphoma subtypes during diagnosis, which can distinguish whether a viral infection is present and does not affect the choice of treatment strategies (8, 10, 11). The present case represented sporadic Burkitt lymphoma, with EBV *in situ* hybridization testing positive.

**Table 1:** Summarization of the cervical Burkitt lymphoma in the past ten years.

Author	Age	Cytomorphology/diagnosis	Treatment	Follow-up months	Outcome
Ceric et al(5)	64	Lymphoma cells are medium in size, with a particularly high mitotic and apoptotic index, with a characteristic starry sky pattern.	Chemotherapy	NA	DOD
de Miranda et al (6)	29	Histopathological report revealed the diagnosis of an extra-nodal BL.	None	1.4	DOD
Cheng et al (7)	47	The patient was diagnosed as primary BL of the uterine cervix at stage II with mutation of TP53 gene, MYC gene and DDX3X gene.	Chemotherapy+Pelvic local radiotherapy	8	DOD
Our case	55	Tumor cells exhibited amosaic-like arrangement, with some cells showing cytoplasmic constriction and well-defined borders.	Chemotherapy	15	AWD

NA, data not available; DOD, died of disease; AWD, alive with disease.

The diagnosis of cervical Burkitt lymphoma is consistent with that of the disease at other sites and is primarily based on histopathological examination, immunohistochemistry, and molecular pathology testing. Histologically, cervical Burkitt lymphoma has an aggressive growth pattern, with tumor cells that are relatively uniform in morphology, of moderate size, and characterized by deeply basophilic cytoplasm often containing lipid vacuoles. Multiple basophilic nucleoli are frequently observed, and mitotic figures are readily identifiable. Aggregates of benign macrophages that phagocytose apoptotic nuclear fragments produce the characteristic “starry sky” phenomenon, which is one of the hallmark histological features (11). Immunophenotypically, the tumor cells exhibit a mature germinal center B-cell profile, with positivity for CD19, CD20, CD79α, PAX5, CD10, and BCL6, and negativity for CD5 and TdT. Among these, a Ki-67 proliferation index approaching 100% is a typical immunophenotypic feature of Burkitt lymphoma (11). In this case, the immunohistochemical results of the tumor cells were consistent with the aforementioned characteristics, such as diffuse CD20 positivity (+), CD10 positivity (+), and Ki-67 positivity (+ >90%), strongly supporting the diagnosis of BL. Genetic alterations: The hallmark genetic abnormality of BL is positive for IG::MYC fusion, with approximately 80% being t(8;14)(q24;q32) translocation, which can lead to the formation of the MYC::IGH fusion gene (10, 11). Additionally, rare t(8;22)(q24;q11) and t(2;8)(p12;q24) translocations may occur, resulting in MYC::IGL and MYC::IGK fusion genes (11). However, it is important to note that MYC translocations are not specific to Burkitt lymphoma and may also be seen in other high-grade B-cell lymphomas (10, 11). In this case, MYC was positive (approximately 80%), and fluorescence *in situ* hybridization (FISH) confirmed IG rearrangements, aligning with the genetic characteristics of Burkitt’s lymphoma and further substantiating the diagnosis.

Differential diagnosis: (a) Diffuse large B-cell lymphoma (DLBCL): Compared to Burkitt lymphoma, DLBCL cells are larger and more irregular in shape. Their immunophenotypic and genetic characteristics also differ, such as a relatively lower incidence of MYC rearrangements and a Ki-67 proliferation index that is generally lower than in BL. Through morphological observation of tumor cells, immunohistochemical staining, and genetic testing, the two can be effectively distinguished (8, 12). (b) High-grade B-cell lymphoma with 11q abnormalities (HGBL-11q): This type of lymphoma exhibits morphological and immunophenotypic characteristics resembling Burkitt lymphoma, but lacks MYC rearrangements. Instead, it harbors characteristic 11q abnormalities. Its pathological morphology and immunophenotype are highly similar to Burkitt’s lymphoma. Therefore, molecular testing is required for diagnosis, with a focus on MYC gene rearrangement, to achieve precise differentiation (8, 11). (c) Nonkeratinizing squamous cell carcinoma of the cervix: Differentiation relies primarily on histopathological and immunohistochemical staining. Cervical non-keratinizing squamous cell carcinoma expresses squamous cell markers, lacks B-cell markers, and does not harbor the genetic alterations typical of Burkitt’s lymphoma (8, 10). By performing immunohistochemical testing on tumor tissues to assess the expression of relevant markers, these two conditions could be effectively distinguished. (d) Neuroendocrine tumors: Neuroendocrine neoplasms exhibit infiltrative growth in nests, trabeculae, or acinar patterns, and are associated with the expression of neuroendocrine markers. Elevated levels of neuroendocrine markers can also be detected in blood (10). The histological morphology and immunophenotype differ substantially from BL, and the two can be distinguished by histopathological HE staining and immunohistochemical staining.

Burkitt lymphoma is characterized by rapid growth and progression. A study by Nasioudis (3) involving 697 female patients with gynecological lymphomas, the five-year survival rate for Burkitt lymphoma of the female reproductive tract was 57%. A case reported in 2018 described a 15-year-old adolescent who was diagnosed with cervical Burkitt lymphoma, and the tumor rapidly shrank following cyclical chemotherapy (13).

Primary cervical Burkitt’s lymphoma is extremely rare and often presents with abnormal vaginal bleeding. Some patients may also experience symptoms, such as abdominal pain or palpable abdominal masses, whereas others may exhibit systemic symptoms, such as fever, night sweats, or weight loss, although these symptoms are relatively atypical. On gynecological examinations, cervical lesions are usually friable, prone to bleeding, and rapidly progressive. Therefore, once diagnosed, a scientific and reasonable treatment plan should be established as soon as possible to improve the patient’s prognosis.

## Data Availability

The original contributions presented in the study are included in the article/supplementary material, further inquiries can be directed to the corresponding author/s.
